# Effect of High-Energy Excimer Treatment of Ti-Based Alloys on Cytocompatibility and Antibacterial Properties

**DOI:** 10.3390/ijms27167250

**Published:** 2026-08-14

**Authors:** Petr Slepička, Silvie Rimpelová, Šárka Havlíčková, Tomáš Kovářík, Jiří Martan, Michal Procházka, Petr Sajdl, Nikola Slepičková Kasálková

**Affiliations:** 1 Department of Solid State Engineering, The University of Chemistry and Technology Prague, 166 28 Prague, Czech Republic; 2 Department of Biochemistry and Microbiology, University of Chemistry and Technology Prague, 166 28 Prague, Czech Republic; 3 New Technologies—Research Centre, University of West Bohemia, Univerzitní 8, 301 00 Plzeň, Czech Republic; 4 Department of Power Engineering, University of Chemistry and Technology Prague, 166 28 Prague, Czech Republic

**Keywords:** titanium, TiAlV, surface enhancement, surface nanopattern, micropattern, morphology, excimer laser, tissue engineering, antibacterial properties

## Abstract

The study investigates the effects of high-energy laser treatment on titanium-based alloys, TiAlV, TiNbZr, and TiNbSnTa, materials of high interest for medical applications such as implants and dental devices due to their exceptional strength-to-weight ratio, corrosion resistance, and biocompatibility. In this research, a unique high-energy laser was used for Ti-based surface activation. The laser exposure induced significant changes in both surface morphology and chemistry while preserving the bulk properties of the substrate. The modified surfaces were evaluated for their impact on cytocompatibility and antibacterial activity. It was found that viability of U-2 OS cells incubated with laser-treated Ti-based substrates was not negatively affected and was comparable to or slightly higher than that of control samples, indicating very good cytocompatibility of the prepared materials. Further, antibacterial evaluation against *E. coli* and *S. epidermidis* demonstrated that laser-treated samples had improved activity, especially against *S. epidermidis*, relative to untreated controls. Thus, these results demonstrate that high-energy laser treatment can simultaneously enhance the biocompatibility and antibacterial properties of titanium alloys, highlighting its potential as a versatile surface modification strategy for advanced biomedical devices.

## 1. Introduction

Laser surface modification is a versatile and controllable approach for tailoring the physicochemical and biological properties of biomedical materials. By adjusting the laser parameters, surface topography, chemistry, and microstructure can be modified from the micrometer to the nanometer scale with limited effects on the bulk material. Laser processing can also create hierarchical structures that influence protein adsorption, cell–material interactions, and bacterial adhesion, making it attractive for implant and tissue-engineering applications. Titanium and its alloys are widely used in orthopedic, dental, and craniofacial implants because of their favorable mechanical properties, corrosion resistance, and biocompatibility. Nevertheless, untreated titanium surfaces are relatively bioinert and may not optimally support cell adhesion, proliferation, and differentiation [1,2]. Surface modification is therefore required to improve osseointegration and reduce the risk of implant-associated infection. For example, Pattanayak et al. [3] used selective laser melting to fabricate bioactive and antimicrobial titanium structures with hierarchical macro-, micro-, and nanoscale porosity, whereas Yan et al. [4] applied one-step micro-arc oxidation to create multifunctional surfaces on additively manufactured titanium implants.

Laser irradiation addresses these limitations by modifying the outermost surface layer through controlled melting, ablation, resolidification, oxidation, grain restructuring, or periodic pattern formation [5]. Laser texturing of titanium surfaces, particularly Ti6Al4V, has been shown to enhance cellular adhesion and viability, including human gingival fibroblast adhesion, while reducing bacterial attachment and colonization [6]. Titanium and β-titanium alloys therefore remain important candidates for laser-assisted surface functionalization [7]. The resulting surface characteristics depend on the laser wavelength, pulse duration, fluence, repetition rate, and processing atmosphere [8]. Appropriate combinations of these parameters can produce controlled oxidation, surface melting, ablation, or laser-induced periodic surface structures (LIPSSs) [9]. The generated structures range from micrometer-scale features that promote mechanical interlocking to nanoscale patterns that affect protein adsorption and cellular signaling [10,11,12,13,14,15]. Femtosecond and nanosecond lasers additionally enable rapid surface patterning with relatively limited heat-affected regions [1]. In particular, LIPSSs can promote osteoblast adhesion while restricting bacterial colonization [2]. Laser- or plasma-induced oxidation may also produce hydrophilic and corrosion-resistant titanium oxide layers that improve early interactions with adhesive proteins [16].

The biological response to laser-modified titanium is governed by the combined effects of surface morphology, roughness, wettability, chemistry, and mechanical properties [17]. Enhanced adhesion and proliferation of osteoblasts, mesenchymal stem cells [18], and fibroblasts have been reported on laser-textured surfaces. Laser-induced micro- and nanostructures can modify the adsorption and conformation of proteins, thereby promoting focal-adhesion formation, cytoskeletal organization, and favorable cell signaling. Endothelial and epithelial cells on laser-functionalized titanium have likewise shown improved spreading and metabolic activity and reduced inflammatory signaling [19]. Laser processing also offers considerable potential for introducing antibacterial functionality [20,21]. Bioinspired antibacterial surfaces and their underlying mechanisms were comprehensively reviewed by Zhang et al. [22]. Laser-generated features may reduce bacterial adhesion, mechanically damage bacterial envelopes, or inhibit biofilm development. These effects can be strengthened by incorporating carbon-based coatings [23], MXenes [24], or metallic nanoparticles such as Ag [25,26,27], whose antibacterial activity may be further enhanced by laser treatment [28]. Several laser-based strategies have combined antibacterial activity with acceptable cytocompatibility. Li et al. [28] improved both properties in a laser-modified Ti-20Zr-10Nb-4Ta alloy. Zhang et al. [29] produced a laser-induced Ti_2_Cu precipitate layer on Ti-13Nb-13Zr-5Cu, achieving a favorable combination of elastic modulus, antibacterial activity, corrosion resistance, hardness, and cytocompatibility. Laser-patterned micro- and nanostructures can also act as reservoirs for Ag nanoparticles, producing temporary anchorage devices with both osteogenic and antibacterial functions [30].

Antibacterial activity may also be achieved by laser texturing without antibacterial additives, as demonstrated by Papa et al. [31]. Alternatively, laser patterning can be combined with hydroxyapatite deposition on Ti-based metallic glasses [32]. In this approach, nanosecond-laser-generated bone-mimetic micropatterns supported hydroxyapatite coatings with different morphologies [32]. Direct femtosecond-laser processing has similarly been used to generate hierarchical LIPSS that promote osteogenesis and osseointegration [33]. Chan et al. [34] reported that treatment of commercially pure Ti and Ti6Al4V with a continuous-wave fiber laser at 1064 nm in a nitrogen-containing atmosphere reduced bacterial adhesion and produced a bactericidal effect. Q-switched nanosecond-laser processing can additionally generate micro/nanostructures with strongly altered wettability, including highly hydrophobic Ti6Al4V surfaces [35]. Laser treatment may also be integrated with chemical or coating-based modifications. Direct laser writing can promote the deposition of biogenic hydroxyapatite and alter the surface chemistry and wettability of Ti6Al4V [36]. Laser-generated micro/nanostructures have been combined with biomimetic peptide grafting to improve interactions with human bone-marrow mesenchymal stem cells [37]. Chemical pseudo-dealloying of Ti-based amorphous alloy ribbons in NH_4_OH/H_2_O_2_ produced nanostructured surfaces with improved cytocompatibility toward Saos-2 and HfOb cells and antibacterial activity against *Pseudomonas aeruginosa* [38]. Nitriding can likewise produce TiZrNb coatings with improved mechanical and electrochemical properties on Ti6Al4V [39]. Rajabi et al. [40] combined nanosecond-laser texturing with electrospun vancomycin-loaded polyvinyl alcohol fibers, obtaining an antibacterial surface without compromising cell viability.

Despite this extensive research, a clear knowledge gap remains. Previous studies have demonstrated that laser irradiation can modify the morphology, chemistry, wettability, cytocompatibility, and antibacterial behavior of commercially pure titanium and TiAlV [41,42,43,44]. However, most investigations have examined only one alloy and have used different laser sources, irradiation parameters, and biological protocols. Excimer laser studies have similarly concentrated mainly on TiAlV or selected model β-titanium alloys and have often focused on morphological or physicochemical changes rather than the combined cytocompatibility and antibacterial response [45,46]. Consequently, it remains unclear whether one set of high-energy excimer laser conditions can generate beneficial surface modifications across compositionally and structurally different titanium alloys while maintaining mammalian-cell viability and suppressing both Gram-negative and Gram-positive bacteria. Direct comparisons of TiAlV, TiNbZr, and the more compositionally complex TiNbSnTa alloy under identical irradiation and biological testing conditions are particularly scarce. Thus, the relationships among alloy composition, laser-induced morphology, surface oxidation or elemental redistribution, and the resulting cellular and bacterial responses have not yet been systematically established [41,42,43,44,45,46].

High-energy excimer irradiation was selected because its ultraviolet radiation is absorbed within a shallow near-surface region of titanium alloys. Rapid energy deposition can therefore induce local heating, melting, ablation, and resolidification while restricting the depth and duration of thermal exposure [45,46]. Compared with longer-wavelength laser processing, the short UV wavelength provides highly localized energy delivery and high spatial resolution. It is also a dry, contact-free, potentially scalable process that does not require chemical etchants, antibacterial dopants, or externally deposited coatings. The comparison of TiAlV, TiNbZr, and TiNbSnTa is scientifically motivated by their different alloying elements and phase constitutions, which may influence both laser–material interactions and the composition of the newly formed surface. TiAlV serves as a clinically established reference, whereas TiNbZr and TiNbSnTa represent advanced alloy concepts containing elements with generally favorable biological profiles [47,48,49]. Nb acts as a β-phase stabilizer, while Zr and Sn contribute to the control of phase stability and mechanical properties; Ta additionally provides high chemical stability and corrosion resistance. These alloying elements may also contribute to the formation of chemically stable surface oxides. Nevertheless, compositional and structural differences can affect optical absorption, heat transfer, melting, elemental diffusion, oxidation, and resolidification, thereby producing alloy-specific surface morphologies and chemical compositions. Applying identical laser conditions to all three alloys enables general effects of high-energy excimer activation to be distinguished from composition-dependent responses.

The principal novelty of this study is the systematic side-by-side comparison of high-energy excimer laser modification of TiAlV, TiNbZr, and TiNbSnTa under identical processing and biological testing conditions. Unlike previous investigations, which have predominantly focused on commercially pure Ti or TiAlV [41,42,43,44], the present work correlates laser-induced changes in surface morphology and chemistry with both mammalian-cell response and antibacterial performance. It therefore evaluates whether a single, one-step, additive-free treatment can provide a favorable balance between cytocompatibility and bacterial suppression across three compositionally different titanium alloys. Cytocompatibility is assessed using human U-2 OS osteoblast-like cells, while antibacterial performance is evaluated against *Escherichia coli* and *Staphylococcus epidermidis* as representative Gram-negative and Gram-positive bacteria, respectively. Including both bacterial groups is important because their different cell-envelope structures may lead to different responses to laser-induced surface features and chemistry. *S. epidermidis* is particularly relevant because of its ability to adhere to artificial surfaces and form persistent biofilms associated with medical-device and implant infections [50]. Accordingly, this study aims to determine how high-energy excimer irradiation modifies the surface morphology and chemistry of TiAlV, TiNbZr, and TiNbSnTa and whether these changes enhance biological functionality without compromising cell viability. The output of the paper may establish high-energy excimer irradiation as a versatile, additive-free, one-step strategy for functionalizing both established and emerging titanium alloys for biomedical implants.

## 2. Results and Discussion

### 2.1. Scanning Electron Microscopy

The morphological changes in the substrate surfaces before and after exposure to a high-energy excimer laser were investigated for TiAlV (TiGr5 ELI), TiNbSnTa, and TiNbZr alloys. High-power laser treatments are commonly employed to fabricate defined surface structures on Ti-based substrates, often affecting relatively deep regions of the material [51] or requiring the application coatings or surface layers [52]. In contrast, surface modification strategies that alter only the outermost layer while preserving the bulk properties of Ti alloys are highly desirable. In this study, a high-energy excimer laser treatment was employed to modify the surfaces of Ti-based materials. Conventional excimer laser processing is typically performed at fluences below 100 mJ·cm^−2^ or even lower, where it is used for construction of laser-induced periodic surface structures (LIPSS) on polymeric materials, particularly aromatic polymers [53]. However, with these laser types/wavelengths, it is generally insufficient to induce pronounced physicochemical changes on Ti-based substrates due to their high melting temperature and thermal stability.

**Figure 1:**
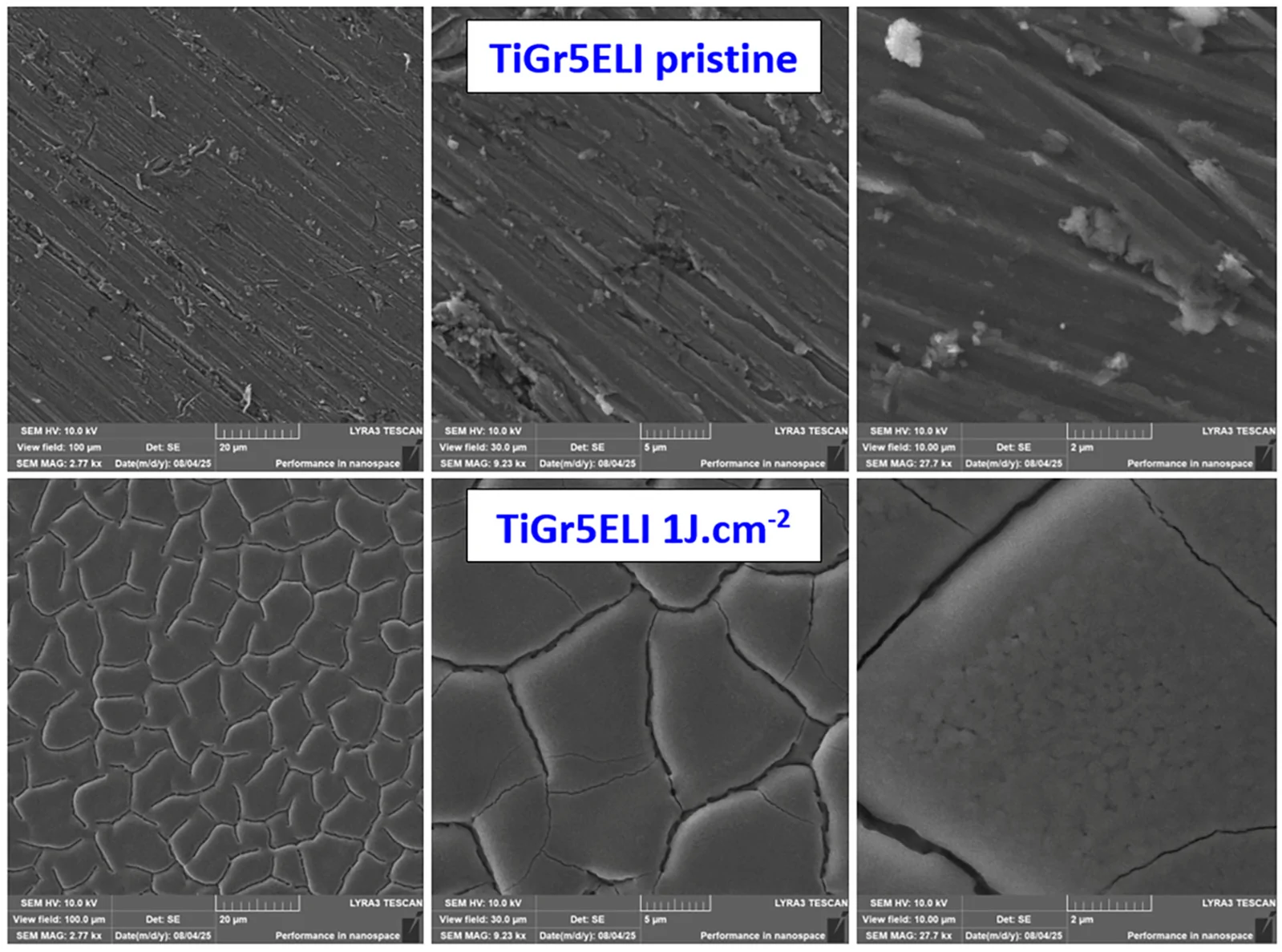
SEM images of pristine and laser-treated TiGr5 ELI substrate. The laser treatment was performed using high-energy laser exposure with 1 J·cm^−2^ with 200 pulses. The scanned areas were of 100 × 100, 30 × 30, and 10 × 10 μm^2^.

Here, a unique high-energy excimer laser system capable of delivering fluences exceeding 1000 mJ·cm^−2^ over relatively large surface areas was utilized. This system has previously been applied for post-processing and remelting of printed periodic structures in TiAl6V4 alloys [54], for which excimer laser treatment effectively removed loosely sintered particles, thereby reducing the risk of particle detachment in in vivo applications and improving implant reliability. A similar surface-confined modification approach was applied to the Ti-based substrates investigated in the present study. Representative SEM images illustrating the laser-induced surface morphology are shown in Figure 1.

The surface of pristine TiGr5 ELI exhibited a lamellar microstructure with apparent grooves of varying shapes and surface inhomogeneities. For surface modification, a laser fluence of 1000 mJ·cm^−2^ combined with 200 laser pulses was applied. These irradiation parameters and the corresponding integral fluence dose were selected based on a preliminary study involving different laser fluences and pulse numbers. Pronounced changes in surface morphology were observed following laser treatment, as documented by the SEM images shown in Figure 1 (bottom row). Laser-induced surface remelting led to the formation of clustered structures separated with troughs. Higher-magnification imaging further revealed a hierarchical morphology, with superimposed micro- and nanoscale features within individual clusters.

**Figure 2:**
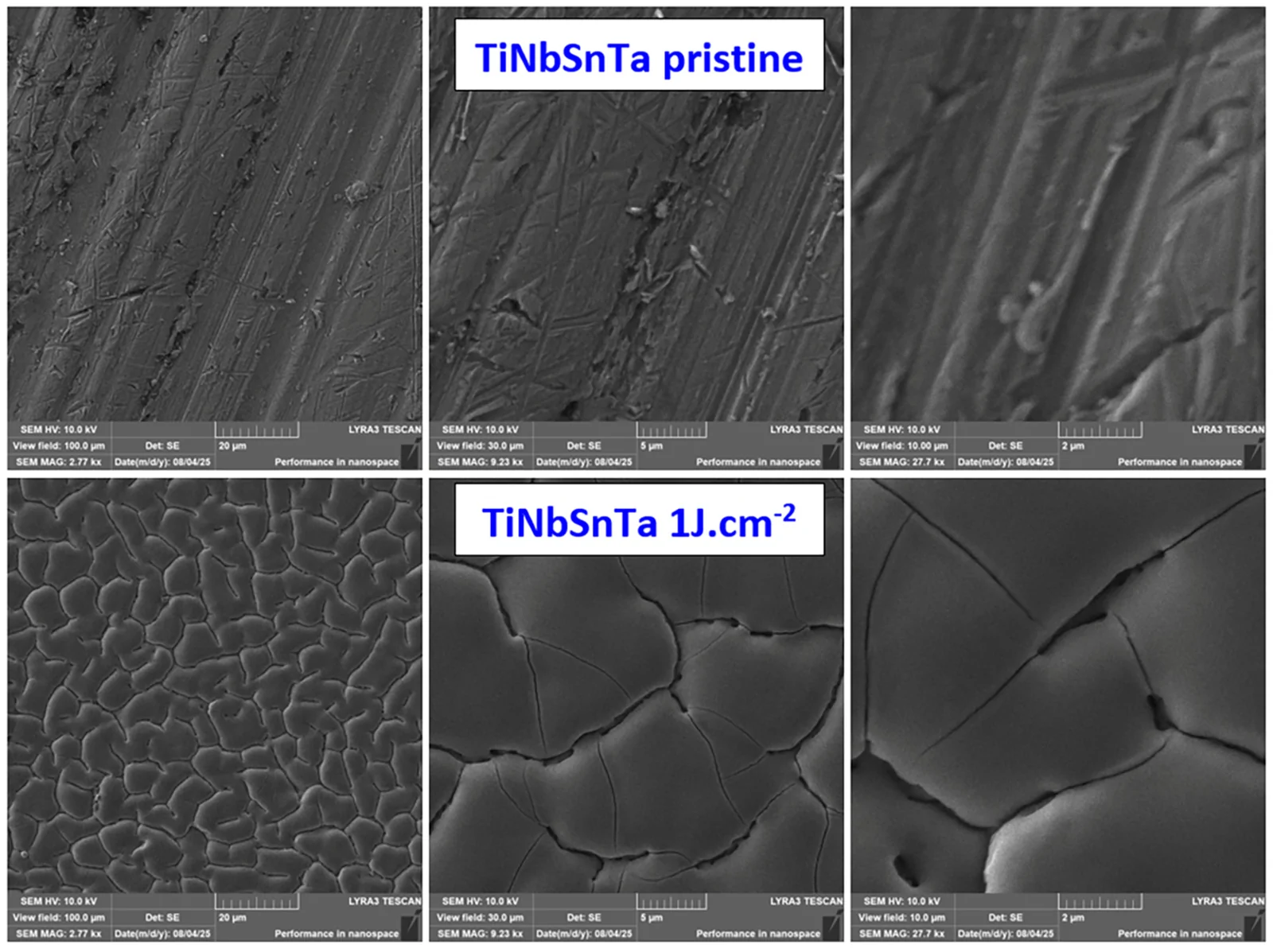
SEM images of pristine TiNbSnTa and this substrate after high-energy laser treatment with 1 J·cm^−2^ and 200 pulses. The scanned areas were of 100 × 100, 30 × 30, and 10 × 10 μm^2^.

**Figure 3:**
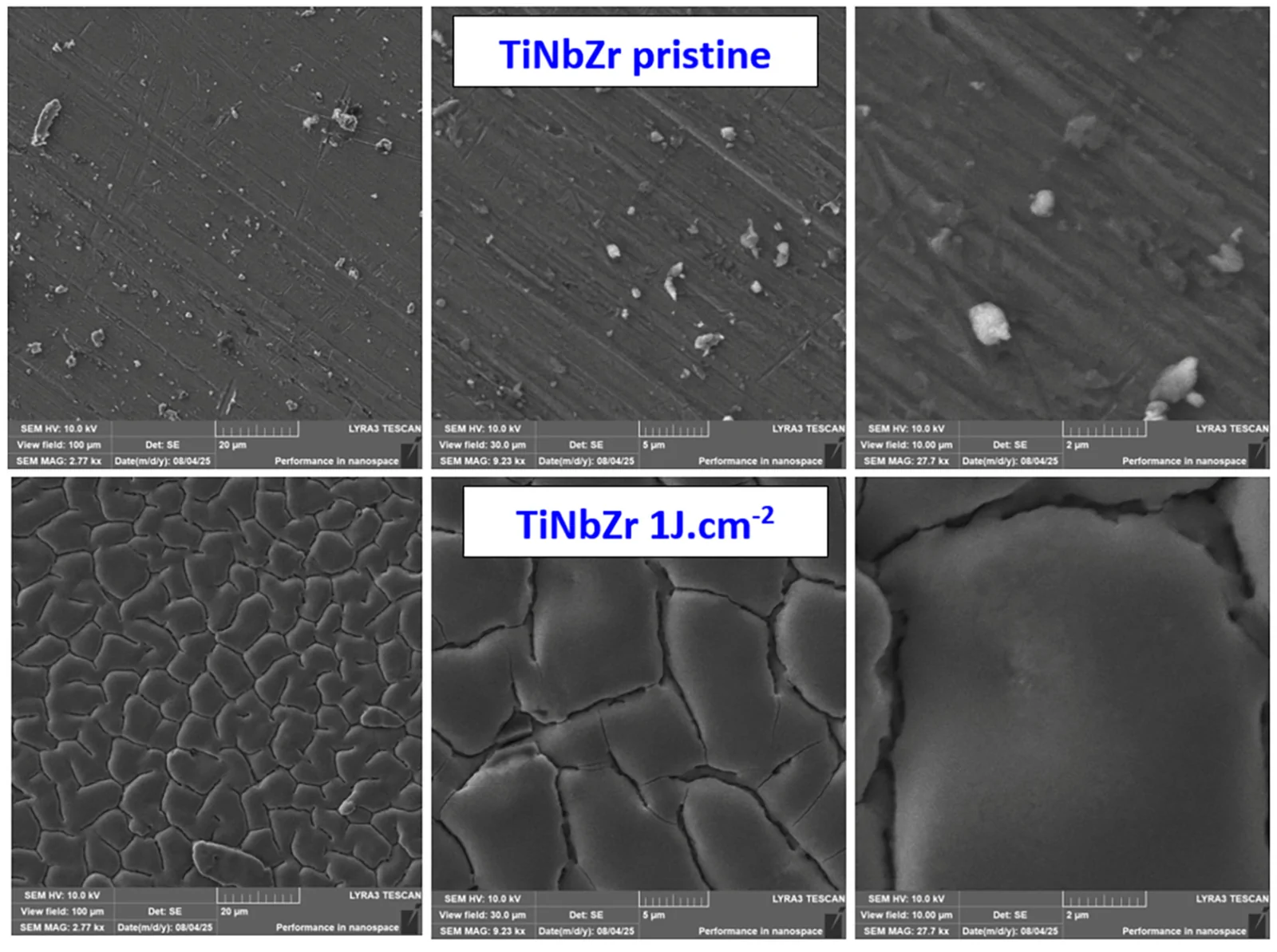
SEM images of pristine TiNbZr and this substrate after high-energy laser treatment with 1 J·cm^−2^ and 200 pulses. The scanned areas were of 100 × 100, 30 × 30, and 10 × 10 μm^2^.

Nanocluster surface features were formed on all laser-exposed samples. As shown in Figure 2, a similar modification of surface morphology was also observed for the TiNbSnTa samples, at which the formation of larger surface clusters was clearly visible on the laser-treated substrates. This surface-selective energy deposition is particularly important for tissue engineering applications, as it ensures that the mechanical properties of the modified substrates remain comparable to those of the pristine materials. In comparison with TiGr5 ELI, laser irradiation of the TiNbSnTa substrate resulted in the formation of smaller surface clusters. Quantitative differences in surface roughness, effective surface area, and surface chemistry will be discussed in subsequent sections based on AFM and EDS analyses. The results of the SEM analysis of TiNbZr samples are introduced in Figure 3, revealing laser-induced surface patterns that are highly similar to those observed for TiGr5 ELI in Figure 1 and Figure 2.

### 2.2. Atomic Force Microscopy

**Figure 4:**
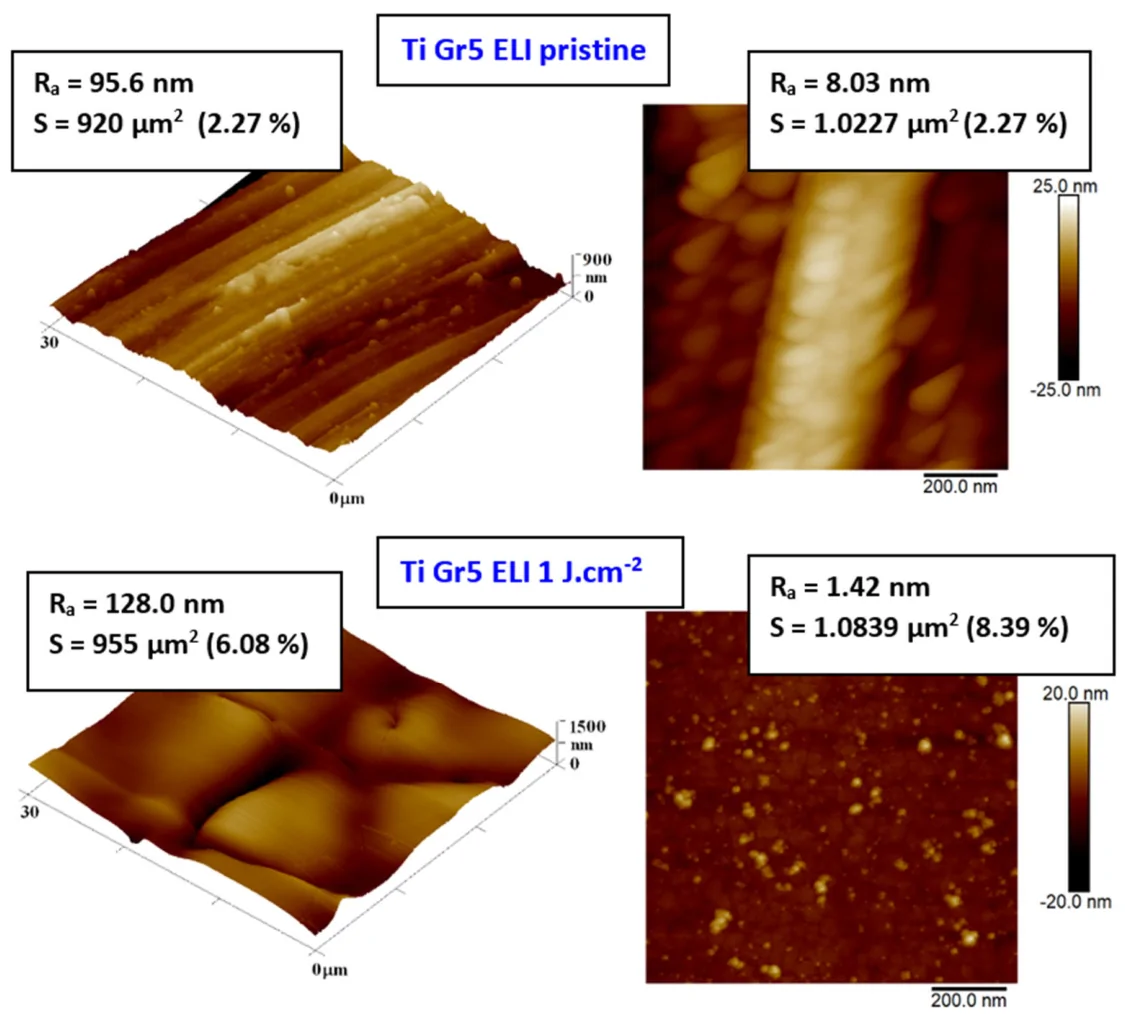
AFM images of pristine TiGr5 ELI and its form treated with high-energy laser treatment with 1 J·cm^−2^ and 200 pulses. The measured areas were of 30 × 30 and 1 × 1 μm^2^. R_a_ represents average surface roughness in nm; S represents an effective surface area (with difference compared to base area in %).

The surface morphology of TiGr5 ELI, TiNbSnTa, and TiNbZr foils before and after excimer laser exposure was analyzed using AFM. Figure 4 shows representative AFM images of pristine TiGr5 ELI substrates and samples treated with a laser fluence of 1000 mJ·cm^−2^ and 200 pulses. A key advantage of AFM analysis is that, in addition to quantitative evaluation of surface roughness and effective surface area, it enables detailed visualization of surface morphology across multiple length scales.

Consistent with the results from the SEM analysis, the AFM analysis of laser-treated TiGr5 ELI samples revealed pronounced cluster formation following excimer laser exposure. We have chosen the presentation of larger 30-micron scans and detailed scans of 1 × 1 μm^2^. Exposure of the samples to the excimer laser “cleaned” the surface, and due to the melting of the Ti-based substrate surface, a nanoclustered pattern was induced and superposed on the original microdomain structure, in agreement with data in Figure 1. High-resolution AFM imaging (Figure 4, bottom right) revealed a nanocluster pattern with an average diameter of ca. 50 nm, together with larger globular nanostructures, which appear as brighter features in the AFM topography. The formation of this micropattern is accompanied by an increase in surface roughness, from ca. 96 nm for pristine samples to 128 nm after laser treatment. This morphological modification also led to an increase in the effective surface area from 2.2% to 6.1% (30 × 30 μm^2^). Such changes in surface topography are expected to positively influence the cytocompatibility of such a material, as discussed in subsequent sections.

**Figure 5:**
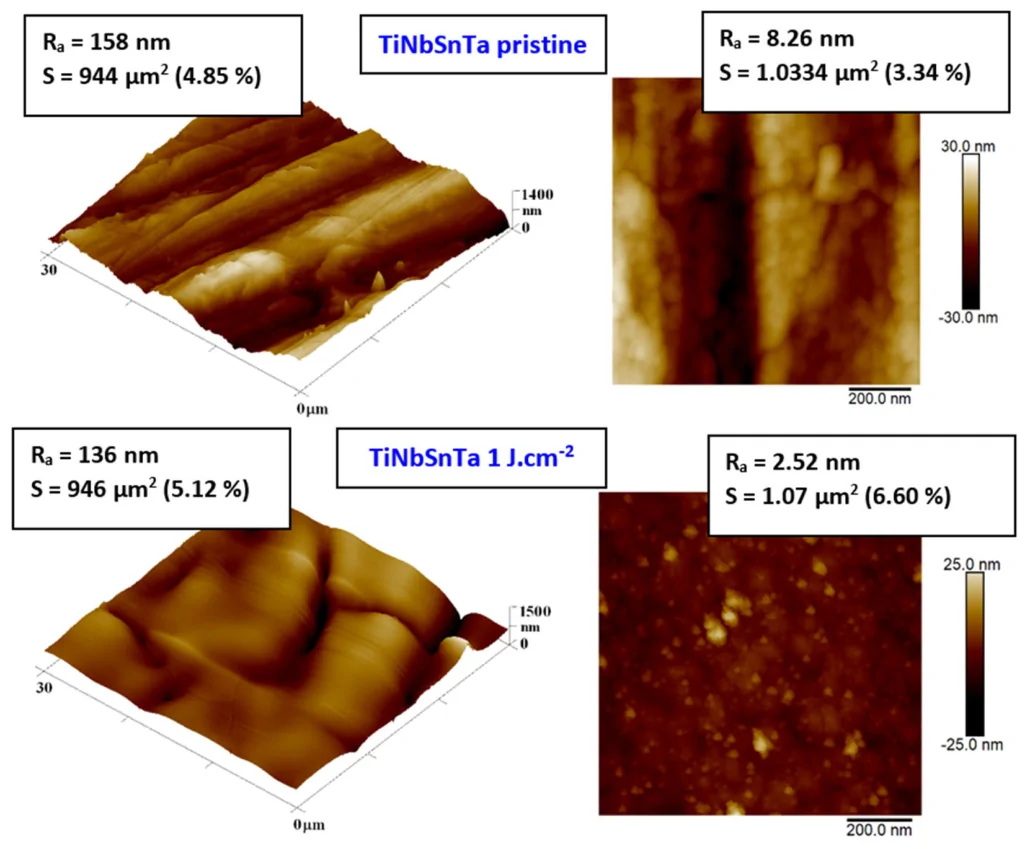
AFM images of pristine TiNbSnTa and this substrate after high-energy laser treatment with 1 J·cm^−2^ and 200 pulses. The measured areas were of 30 × 30 and 1 × 1 μm^2^. R_a_ represents average surface roughness in nm; S represents an effective surface area (with difference compared to base area in %).

**Figure 6:**
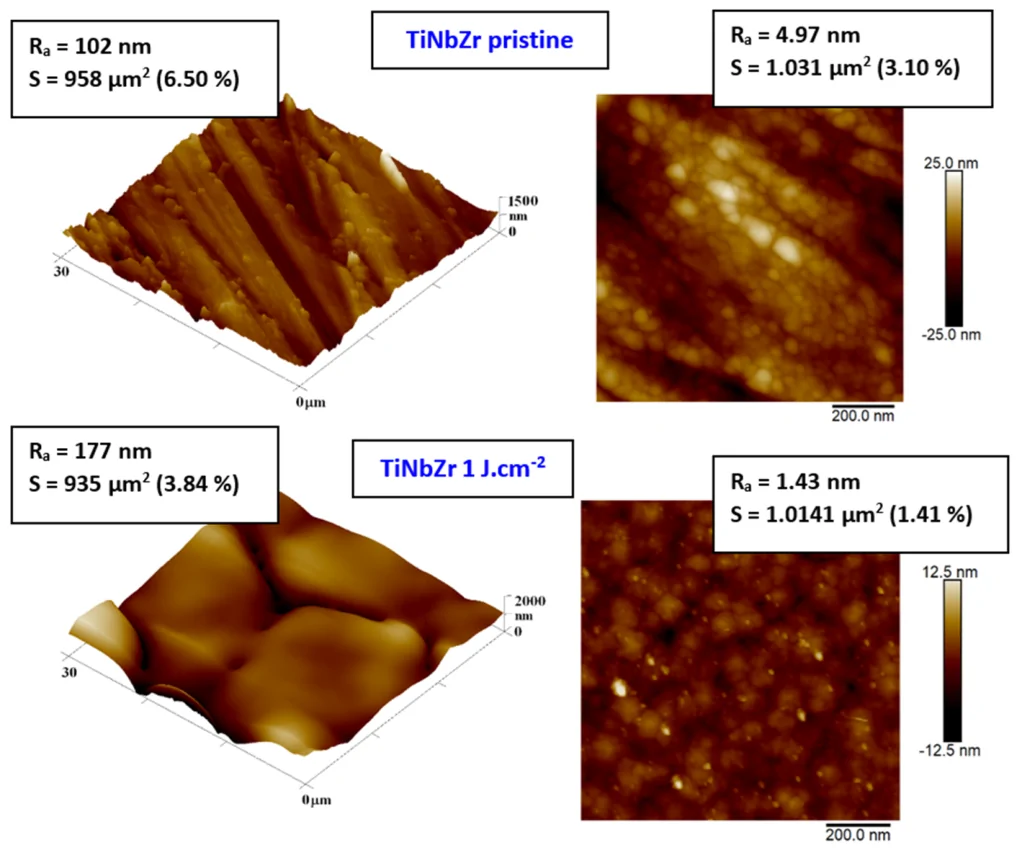
AFM images of pristine TiNbZr and this substrate after high-energy laser treatment with 1 J·cm^−2^ and 200 pulses. The measured areas were of 30 × 30 and 1 × 1 μm^2^. R_a_ represents average surface roughness in nm; S represents an effective surface area (with difference compared to base area in %).

Changes in surface morphology, detailed surface structure, surface roughness, and effective surface area were also determined for TiNbSnTa and TiNbZr (Figure 5 and Figure 6, respectively) samples using AFM analysis. Comparison of the pristine samples in Figure 4 and Figure 5 reveals distinct differences between TiGr5 ELI and TiNbSnTa. While both materials exhibited lamellar surface morphology prior to excimer laser treatment, the pristine TiNbSnTa substrate showed significantly higher roughness than TiGr5 ELI. This increased roughness can be attributed to the more pronounced wavy topography present on the surface of pristine TiNbSnTa. Similar to the TiGr5 samples, laser irradiation induced a superimposed nanopattern, which is clearly visible in the high-resolution AFM image shown in Figure 5 (bottom right). In contrast, excimer laser treatment of TiNbZr substrates resulted in a marked increase in surface roughness. A substantial rise from 102 nm to 177 nm was observed, primarily associated with the formation of microdomains, as shown in Figure 6 (bottom row). These results demonstrate that while excimer laser exposure induces comparable hierarchical surface patterning across investigated Ti-based alloys, the extent and direction of roughness modification are strongly dependent on the substrate composition.

The increase in surface roughness, in contrast, is not correlated with effective surface area of the TiNbZr sample. A distinct difference in pristine TiNbZr is also apparent in detail, as only this pristine substrate exhibited a grainy, nanoglobular pattern (upper left image in Figure 6). This feature, however, does not significantly influence the morphology induced by excimer laser exposure. Among the studied Ti-based substrates, the induced microdomain pattern on TiNbZr showed the highest surface roughness (177 nm), exceeding that of TiNbSnTa (136 nm), and TiGr5 ELI (128 nm). These differences in surface roughness and morphology may play an important role in cell attachment and survival.

### 2.3. Energy-Dispersive Spectroscopy

Surface chemical composition plays an important role in surface–cell interactions; therefore, we focused in detail on chemical changes induced on the investigated surfaces by high-energy excimer laser treatment. The pristine samples were analyzed first (first column in Figure S1). As expected, the nominal chemical composition of the pristine materials was confirmed. However, likely due to polishing procedures and subsequent interaction with the ambient atmosphere, oxygen and, in some cases, carbon were detected on the pristine surfaces. Excimer laser treatment induced pronounced changes in surface chemistry. In particular, when carbon was present on the pristine surface, laser exposure led to the effective removal of the residual carbon from the treated samples. The most significant induced chemical change was an increase in surface oxygen content for all investigated substrates: TiGr5 ELI (from 6.2 to 19.6 wt. %), TiNbZr (from 3.8 to 19.6 wt.%), and TiNbSnTa (from 6.2 to 22.1 wt. %). The presence of oxygen confined to this surface-enhanced layer may influence cell–surface interactions [55].

Another notable change was observed for the TiNbSnTa substrate, for which laser exposure resulted in a reduction in the surface concentrations of Sn and Ta. This effect was confirmed by repeated irradiation and subsequent analyses. The phenomenon is most likely caused by diffusion processes within the laser-induced remelted layer, driven by high-energy annealing and elemental diffusion into greater depths of the material. As discussed later, this effect did not adversely affect the cytocompatibility of the treated samples. As a complementary analytical technique for surface chemical characterization, X-ray photoelectron spectroscopy was employed.

### 2.4. XPS Analysis

**Figure 7:**
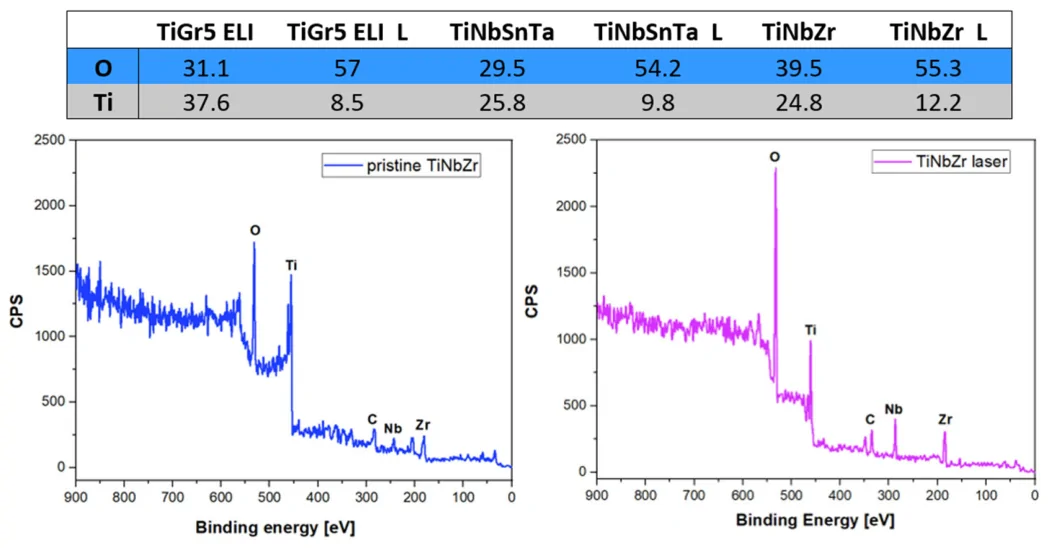
Atomic concentration from XPS spectra of Ti and C for pristine TiGr5 ELI, TiNbZr, and TiNbSnTa and these substrates after high-energy laser exposure with 1 J·cm^−2^ and 200 pulses (upper table). Particular XPS spectra for pristine TiNbZr and exposed TiNbZr with excimer laser 1 J·cm^−2^ and 200 pulses are introduced in the bottom part.

For clarity we present the XPS spectra of the pristine TiNbZr sample alongside those of the sample treated at 1 J·cm^−2^ with 200 pulses. In addition, we report the atomic concentrations of elements that were significantly affected by laser treatment, namely titanium and oxygen (see Figure 7). Consistent with the EDS results, XPS analysis confirmed a substantial increase in oxygen content for all investigated samples following high-energy excimer laser annealing.

This increase in oxygen atomic concentration was accompanied by a pronounced decrease in the titanium concentration at the immediate surface. It should be noted that the XPS technique provides information from only the outermost surface layers of both pristine and treated materials. Consequently, direct comparison of absolute concentration values obtained by XPS and EDS is not straightforward. Nevertheless, both techniques consistently confirm the same trend, namely a significant laser-induced increase in surface oxygen content.

**Figure 8:**
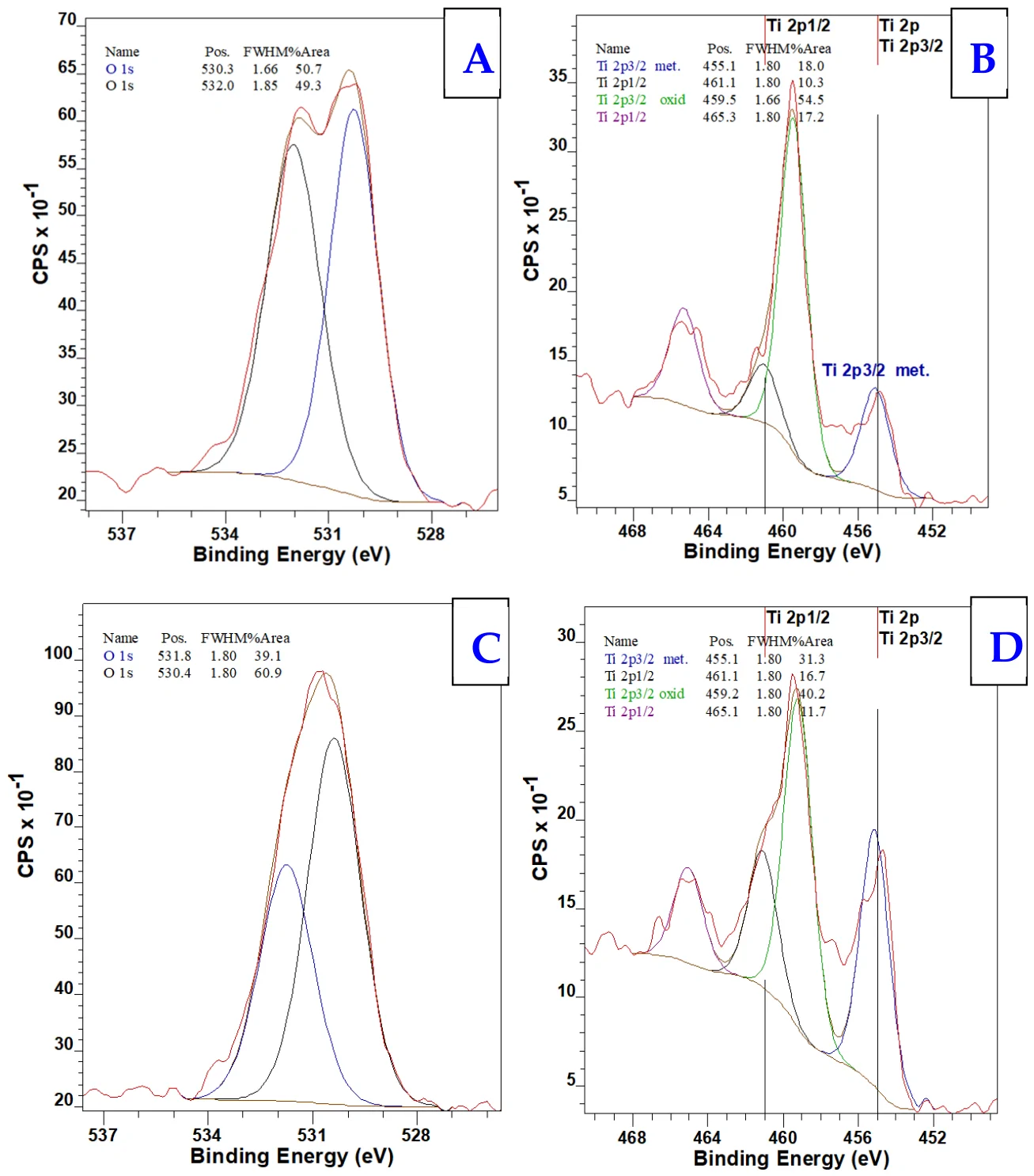
Deconvoluted XPS spectra for pristine TiGr5 ELI—oxygen (**A**) and titanium (**B**)—and the deconvoluted spectra for laser-treated (1000 mJ·cm^−2^) TiGr5 ELI—oxygen (**C**) and titanium (**D**).

For the detailed XPS analysis with peak deconvolution we selected the TiGr5 ELI samples, a pristine one and a laser-treated one, and the analysis was performed without argon cleaning and with an attempt to thermally desorb contaminating hydrocarbons. The deconvoluted O 1s spectrum of pristine Ti Grade 5 (Ti–6Al–4V) contains two nearly equally intense components (Figure 8A), indicating that its native passive layer consists of both lattice oxide and surface-bound oxygen species. The O 1s spectrum of pristine Ti Grade 5 was deconvoluted into two components centered at 530.3 and 532.0 eV. The lower-binding-energy peak at 530.3 eV, and relative area of 50.7%, can be assigned predominantly to lattice O^2−^ ions in the native surface oxide layer. This component is expected to originate mainly from TiO_2_, although overlapping contributions from aluminum and vanadium oxides cannot be excluded because of the alloy composition. The higher-binding-energy component at 532.0 eV represented 49.3% of the total O 1s signal. This peak is attributed primarily to non-lattice oxygen species, particularly surface hydroxyl groups, together with possible contributions from adsorbed water and oxygen-containing adventitious carbon. The nearly equal proportions of lattice and non-lattice oxygen suggest that the naturally formed oxide film is strongly hydroxylated and hydrated following exposure to ambient conditions. Such surface hydroxylation may influence wettability, protein adsorption and subsequent biological interactions of the alloy. The Ti 2p spectrum of pristine Ti Grade 5 (Figure 8B) was deconvoluted into two spin–orbit doublets. These components are characteristic of Ti^4+^ species and can therefore be assigned predominantly to TiO_2_ within the naturally formed passive surface layer. Together, these peaks represent 71.7% of the fitted Ti 2p signal, confirming that oxidized titanium is the major chemical state detected at the alloy surface. The second doublet lower-binding-energy contribution was assigned in the fit to metallic titanium originating from the underlying alloy. Its detection indicates that the native oxide film is sufficiently thin or locally heterogeneous to allow photoelectrons from the subsurface alloy or metal–oxide interface to reach the detector. Together, the Ti 2p and O 1s results indicate a stratified native surface structure consisting of an underlying metallic Ti alloy, a possible reduced TiO_x_ interfacial region and an outer TiO_2_-rich passive film. The spectra indicate that high-energy excimer laser treatment substantially modifies the native passive layer of Ti Grade 5. The main changes are an increased contribution from reduced/metal-like titanium and a lower proportion of hydroxylated or adsorbed oxygen species.

The O 1s spectrum of the laser-treated alloy (Figure 8C) contained two components at 530.4 and 531.8 eV. The peak at 530.4 eV, representing 60.9% of the O 1s envelope, is assigned to lattice O^2−^ ions in titanium and possibly other alloying-element oxides. The component at 531.8 eV, with a relative area of 39.1%, can be attributed to surface hydroxyl groups, low-coordination oxygen and other adsorbed oxygen-containing species. The increase in the relative proportion of lattice oxygen, accompanied by a decrease in the higher-binding-energy O 1s component, suggests laser-induced removal of adsorbed water, surface hydroxyl groups and weakly bound oxygen-containing contaminants. The residual or newly formed oxide therefore appears less hydrated and relatively richer in lattice oxygen than the original native film. The Ti 2p spectrum of laser-treated Ti Grade 5 (Figure 8D) was resolved into two spin–orbit doublets. The higher-binding-energy doublet consisted of two Ti components are assigned predominantly to Ti^4+^ in TiO_2_ and represented 51.9% of the total fitted Ti 2p area. The lower-binding-energy doublet, located at 455.1 and 461.1 eV, accounted for the remaining 48.0% of the Ti 2p signal. This contribution can be attributed to reduced or metal-like titanium species originating from the underlying alloy or from an oxygen-deficient TiO_x_ interfacial layer. Compared with the pristine surface, the relative contribution of the lower-binding-energy titanium component increased from 28.3 to 48.0%, whereas the Ti^4+^ contribution decreased from 71.7 to 51.9%. This pronounced change indicates that high-energy laser irradiation disrupted the original passive oxide film. Rapid laser-induced heating, melting and possible ablation may have partially removed or reduced the native TiO_2_ layer, exposing the underlying alloy or producing reduced titanium oxides during rapid resolidification. High-energy excimer laser irradiation altered both the oxidation state of titanium and the chemical environment of surface oxygen. Compared with the pristine alloy, the laser-treated surface exhibited a substantially higher contribution from reduced or metal-like titanium species and a correspondingly lower Ti^4+^ contribution. Simultaneously, the O 1s spectrum shifted towards a larger relative proportion of lattice oxygen and a lower contribution from hydroxylated and adsorbed species. These results suggest partial disruption or ablation of the native hydrated TiO_2_ film, followed by rapid formation of a less hydroxylated and chemically heterogeneous TiO_x_ layer during resolidification and subsequent air exposure.

### 2.5. Antibacterial Properties

The antibacterial performance of pristine TiGr5 ELI, TiNbZr, and TiNbSnTa substrates and of the corresponding surfaces treated by a high-energy excimer laser was evaluated against *Escherichia coli* and *Staphylococcus epidermidis*, representing Gram-negative and Gram-positive bacteria, respectively. Viable bacteria were quantified as colony-forming units (CFU) after 3 and 6 h of direct contact, and the results were expressed relative to the corresponding time-matched control.

**Figure 9:**
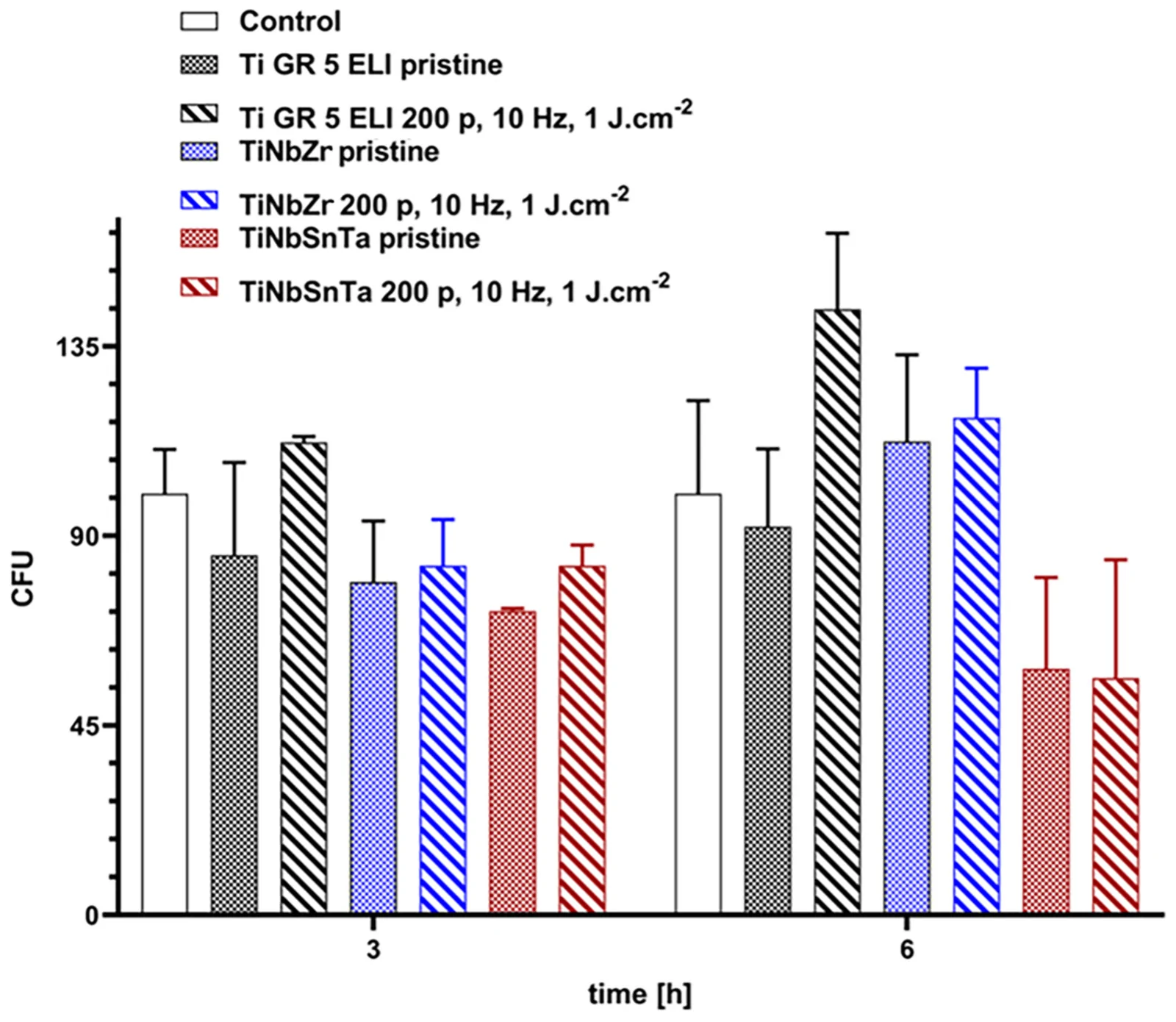
Antibacterial activity of pristine TiGr5 ELI, TiNbZr, and TiNbSnTa and these substrates after high-energy laser treatment with 1 J·cm^−2^ and 200 pulses against *S. epidermidis* after 3 and 6 h of incubation. CFU—number of colony-forming units.

**Figure 10:**
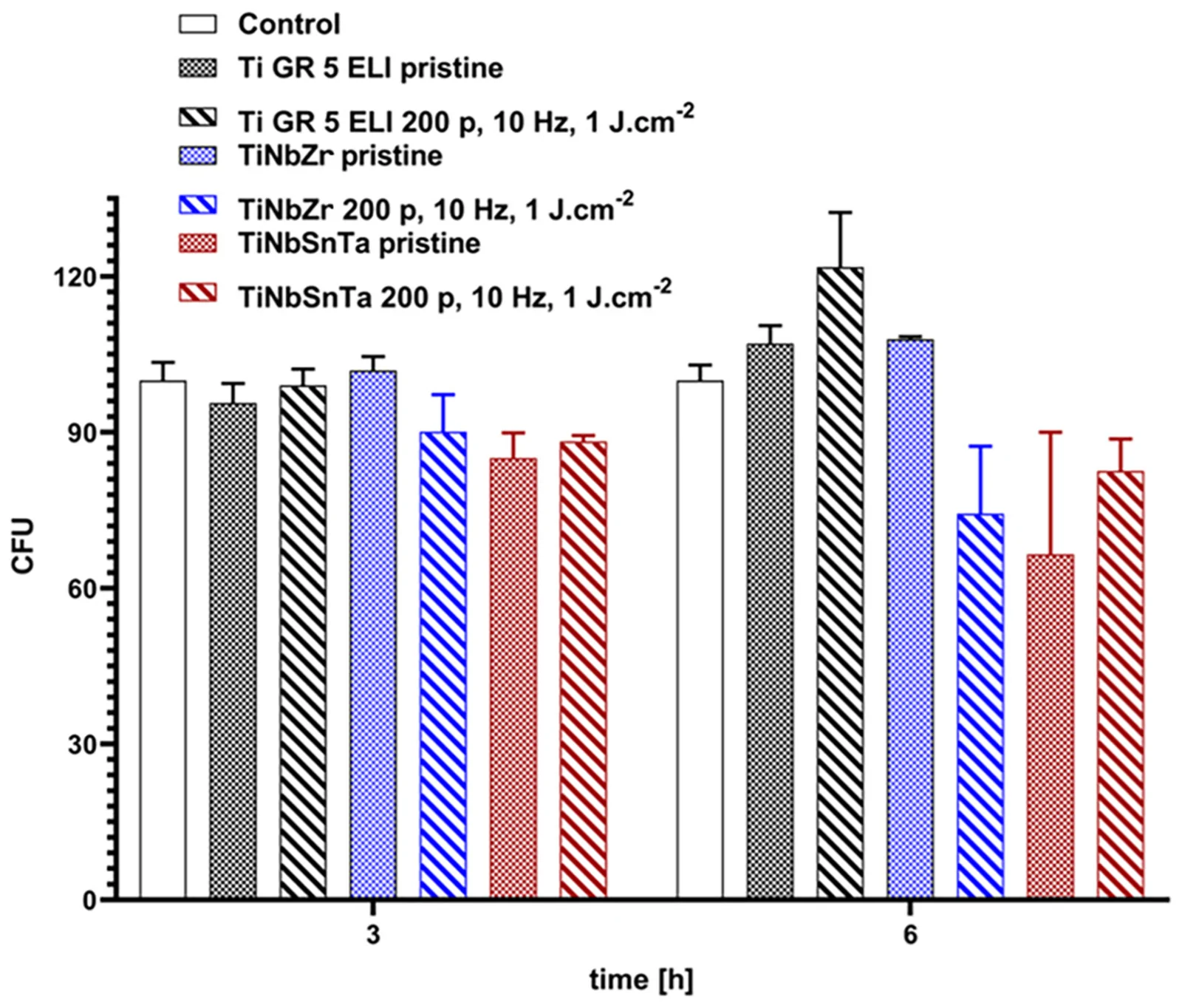
Antibacterial activity of pristine TiGr5 ELI, TiNbZr, and TiNbSnTa and these substrates after high-energy laser treatment with 1 J·cm^−2^ and 200 pulses against *E. coli* after 3 and 6 h of incubation. CFU—number of colony-forming units.

After 3 h, TiNbSnTa was the only alloy that consistently decreased the relative CFU count for both bacterial strains on both the pristine and laser-treated surfaces (Figure 9 and Figure 10). This response persisted after 6 h: both TiNbSnTa surfaces maintained lower CFU counts for *S. epidermidis* and *E. coli* than the respective controls. Laser-treated TiNbZr additionally produced a pronounced decrease in *E. coli* CFU after 6 h, whereas no comparable reduction was observed for *S. epidermidis*. TiGr5 ELI did not exhibit an antibacterial response under the tested conditions, irrespective of laser treatment, and the laser-treated TiGr5 ELI surface showed a numerical increase in CFU recovery after 6 h. Some TiNbZr conditions likewise showed increased *S. epidermidis* counts at the later time point. These findings demonstrate that the biological outcome is governed by an interaction among alloy composition, the surface state generated by laser processing, bacterial strain, and contact time, rather than by laser exposure alone.

The persistence of the TiNbSnTa response on both pristine and laser-treated samples indicates that alloy-dependent surface chemistry is an important component of the antibacterial behavior. Titanium alloys spontaneously develop nanometer-scale passive films, and bacteria therefore interact with a mixed oxide/hydroxide surface rather than directly with the bulk metal. Air-formed films on Ti-Nb-Ta-Zr alloys contain oxidized species derived from Ti, Nb, Ta, and Zr [56], whereas passive films on Ti-Nb-Sn alloys contain TiO_2_, Nb_2_O_5_, and SnO_2_ [57]. Variations in the relative abundance, chemical state, hydroxylation, and spatial distribution of these oxides can modify surface potential, acid–base interactions, wettability, protein or medium conditioning, and the accessibility of attachment sites. The chemically more complex passive layer of TiNbSnTa may therefore contribute to its distinct response. However, the present CFU assay does not distinguish inhibition of adhesion or culturability from direct killing, and it does not establish whether ion release contributes to the effect. Accordingly, the result is most appropriately described as an antibacterial response rather than as proof of a bactericidal mechanism.

Excimer irradiation can further alter the passive film through rapid melting, ablation, resolidification, oxidation, and redistribution of alloying elements, while simultaneously generating new micro- and nanoscale features. The influence of these changes on bacterial attachment is not monotonic: valleys and resolidified asperities may increase the available contact area or provide protected niches, whereas sufficiently fine features may reduce the number of effective attachment points or mechanically stress the bacterial envelope. Experimental studies have consequently shown both enhanced and reduced bacterial retention as a function of roughness, feature geometry, wettability, and oxide chemistry [58,59,60,61,62]. Thus, average roughness or contact angle alone cannot explain the present data. The selective reduction in *E. coli* on laser-treated TiNbZr after 6 h plausibly reflects a TiNbZr-specific combination of oxide composition and feature dimensions that is less favorable for the rod-shaped Gram-negative cells, but not for the smaller coccoid *S. epidermidis*. Such strain specificity is consistent with previous observations that the same laser-generated structure can produce opposite retention trends for Gram-negative and Gram-positive bacteria [62]. This interpretation remains mechanistic but provisional because direct measurements of bacterial attachment, membrane integrity, and surface charge were not included in the present assay.

The numerical increase in CFU recovery after 6 h for laser-treated TiGr5 ELI and for several other alloy–strain combinations can be explained by the time-dependent nature of the assay. The recovered CFU count represents the net result of bacterial survival and multiplication, reversible attachment and detachment, possible retention within surface features, and the efficiency with which viable cells are recovered for plating. When a surface does not provide a sustained inhibitory stimulus, cells that survive the initial contact period may adapt and multiply between 3 and 6 h. In addition, adsorption of constituents from the incubation medium can progressively condition the surface, while microtopographical recesses can provide favorable attachment sites. Importantly, because the data are normalized to a time-matched control, a value above 100% means that more culturable cells were recovered than from the control under the same conditions; it should not, by itself, be interpreted as evidence that the material actively promotes bacterial growth, particularly when error bars overlap and statistical significance has not been established. The later increase therefore supports the conclusion that TiGr5 ELI lacks a durable antibacterial effect under these conditions and that some laser-generated surface states may favor bacterial retention or recovery. From a biological perspective, TiNbSnTa provided the broadest and most persistent early suppression across the two model organisms, whereas the benefit of laser treatment for TiNbZr was restricted to *E. coli* and emerged only after 6 h. High-energy excimer processing should therefore be regarded as an alloy-specific surface-tuning strategy rather than a universally antibacterial treatment.

### 2.6. Cytocompatibility

**Figure 11:**
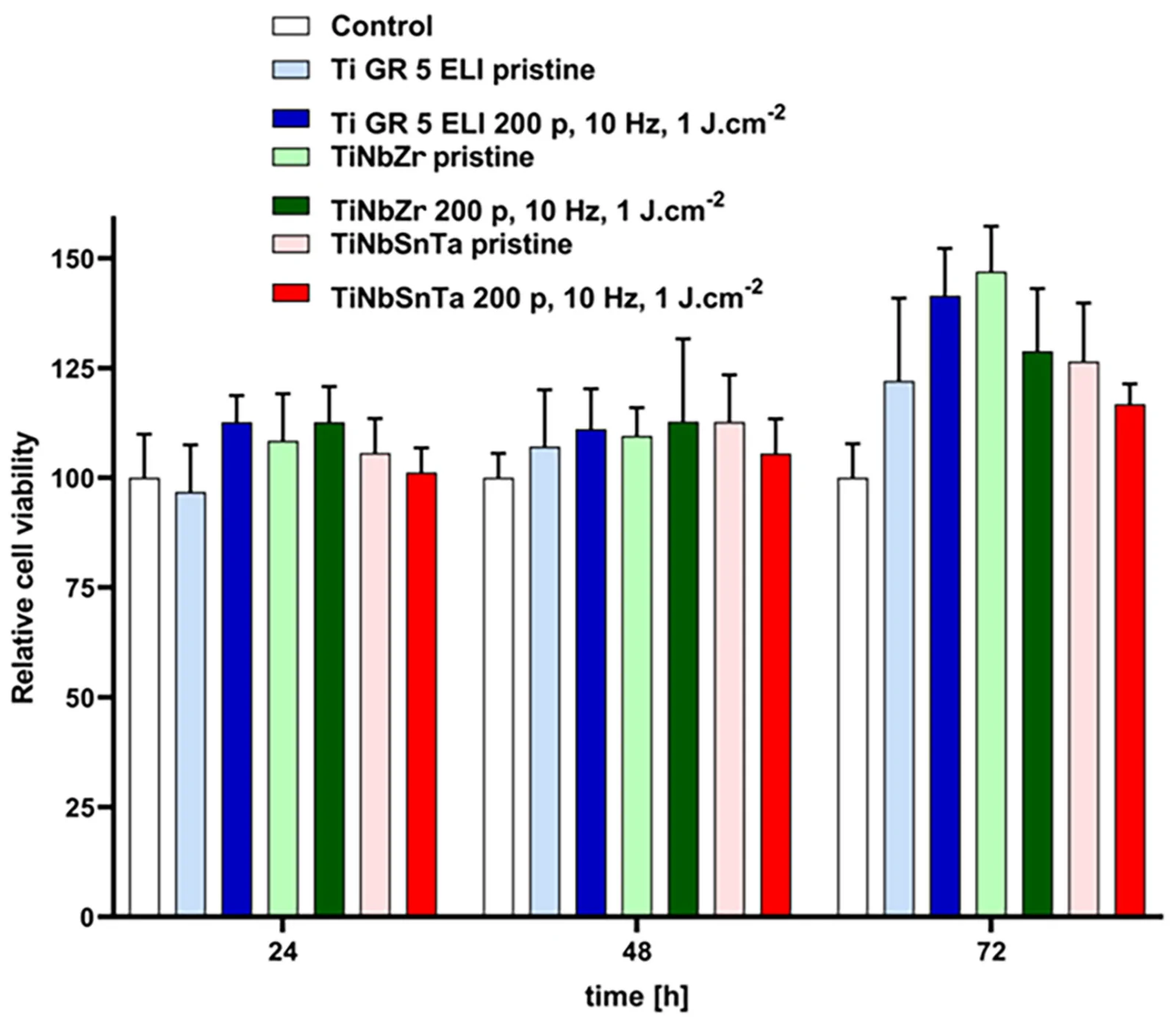
Viability of U-2 OS cells following exposure to leachates obtained from pristine and laser-treated TiGr5 ELI, TiNbZr, and TiNbSnTa substrates, evaluated after 24, 48, and 72 h of incubation.

The cytocompatibility of both pristine and laser-treated TiGr5 ELI, TiNbZr, and TiNbSnTa substrates was evaluated using U-2 OS cells in order to assess the potential effects of substrate-derived leachates on cell viability over time (24–72 h) (Figure 11). Overall, no substantial differences were observed between cells exposed to leachates from any of the tested materials and the control cells cultured in standard cell culture medium alone. This indicates good cytocompatibility of all investigated substrates irrespective of their composition or surface modification.

At 24 and 48 h of incubation, U-2 OS cell viability remained comparable across all pristine and laser-treated samples and did not differ significantly from the control. A slight difference was observed only after prolonged incubation (72 h), when leachates from laser-treated TiGR5 ELI and pristine TiNbZr induced a modest increase in cell viability relative to the control. This enhancement, however, was minor and within a range that does not indicate any adverse effects. Taken together, these results demonstrate that both pristine and laser-treated Ti-based substrates exhibited good cytocompatibility.

The cellular response should be considered in relation to the pronounced reconstruction of the alloy surfaces induced by excimer laser irradiation. Laser treatment transformed the original lamellar or grain-like morphology into a hierarchical surface composed of resolidified microstructures, troughs, and nanoscale clusters, accompanied by an increase in roughness and effective surface area. Such multiscale topographies may provide additional anchoring sites and modify the adsorption and conformation of extracellular-matrix proteins, thereby influencing subsequent integrin-mediated cell adhesion and signaling. Similar effects were reported by Donaghy et al. [63], who observed improved mesenchymal stem-cell spreading and bone-like nodule formation on fiber-laser-treated Ti–Nb–Zr–Ta surfaces containing combined micro- and nanoscale features. Liu et al. [64] likewise demonstrated that hierarchical laser-generated structures enhanced cell proliferation, osteogenic differentiation, mineralization, and collagen secretion. Furthermore, femtosecond-laser modification of Ti6Al4V scaffolds was shown to increase roughness, convert the surface from hydrophobic to superhydrophilic, and promote osteoblast adhesion, differentiation, and bone ingrowth in vivo [65]. Nevertheless, the biological response cannot be attributed to roughness alone; Ferraris et al. [41] obtained biocompatible laser-textured surfaces that guided fibroblast orientation despite a reduction in wettability, emphasizing the combined influence of feature dimensions, organization, and surface chemistry.

The substantial oxygen enrichment detected after laser treatment indicates the formation or thickening of a mixed passive oxide layer. Ti-, Nb-, Zr-, Ta-, and Sn-based oxides are chemically stable and may limit the release of potentially harmful metallic species, while hydroxylation of the oxide surface under physiological conditions can increase its polarity and affect protein adsorption. Redistribution of Sn and Ta within the remelted TiNbSnTa layer may also have modified the composition of this outer oxide without producing cytotoxic leachates. These effects provide a plausible explanation for the preserved, and in some cases slightly increased, U-2 OS viability after 72 h. However, because viability was assessed using substrate leachates, the present results primarily demonstrate the absence of soluble cytotoxic products; they do not directly establish a topography-mediated enhancement of cell adhesion or proliferation. Direct-contact cell studies would therefore be required to confirm the proposed morphology–cell interactions.

All investigated substrates, whether pristine or laser-treated, maintained U-2 OS cell viability comparable to the control, demonstrating that laser-induced changes did not generate cytotoxic leachates. However, antibacterial activity varied markedly with alloy composition and bacterial strain. TiNbSnTa exhibited the most favorable overall biological profile, as both pristine and laser-treated surfaces reduced *S. epidermidis* and *E. coli* counts while maintaining good cytocompatibility. Nevertheless, because antibacterial activity was already present on pristine TiNbSnTa, its performance cannot be attributed exclusively to laser treatment and probably reflects the contribution of alloy composition and surface oxide chemistry. Laser-treated TiNbZr also showed a promising balance, although its antibacterial effect was delayed and observed mainly against *E. coli* after 6 h. In contrast, TiGr5 ELI remained cytocompatible but did not significantly inhibit either bacterial strain, indicating that favorable mammalian-cell compatibility does not necessarily coincide with antibacterial activity. Importantly, none of the antibacterial responses was accompanied by decreased U-2 OS viability, suggesting that bacterial inhibition was achieved without an evident cytotoxic trade-off under the applied experimental conditions. Among the investigated materials, laser-treated TiNbSnTa therefore appears to provide the most favorable balance between broad antibacterial activity and cellular safety, while laser-treated TiNbZr represents a more strain-specific alternative.

## 3. Materials and Methods

### 3.1. Materials

For the experiments, the following substrates were used: TiGr5 ELI (Titanium Grade 5 Extra Low Interstitial, Ti6Al4V), TiNbZr, and TiNbSnTa. The samples were supplied by New Technologies-Research Centre at the University of West Bohemia (Plzeň, Czech Republic). The thickness of the samples was 1.2 mm. The samples were rounded in shape with a diameter of 1 cm. Surface polishing of the samples was applied.

### 3.2. Excimer Laser Treatment

A high-power excimer KrF laser (Coherent Inc., Santa Clara, CA, USA, Leap 100 K) was used with a wavelength of 248 nm, a pulse duration of 20–40 ns, an output energy of up to 1000 mJ, and beam size with an aperture of 32 × 13 mm^2^. In all cases, the laser beam acted parallel to the normal of the sample surface (at an angle of 0°). The energy of the laser beam was 1000 mJ·cm^−2^, the number of laser pulses used ranged between a single shot and 500 pulses, and finally 200 pulses was selected. The repetition rate of the laser exposure was set to 10 Hz. The optimization of the laser paremeters was carried out previously, when we studied the cytocompatibility of carbon-coated Ti alloys [23], but we updated the optimization procedure significantly for this experiment. We first applied a laser fluence of 100 mJ·cm^−2^ while using 200 pulses. This exposure did not change the surface morphology of the treated foils. The laser fluence was further increased continuously by 100 mJ·cm^−2^ at each step (100, 200…etc.). When we reached a fluence of 500 mJ·cm^−2^, we observed some morphological changes. We further increased the laser fluence up to 1200 mJ·cm^−2^. By comparison of this set, mainly by LCM and partially by AFM analysis, we selected 1000 mJ·cm^−2^ for the series of our experiments.

### 3.3. Analytical Methods

#### 3.3.1. Scanning Electron Microscopy

The morphology of the sample surfaces was also characterized by a complementary technique using the scanning electron microscope, FIB-SEM LYRA3 GMU (Tescan. Brno, Czech Republic). The acceleration voltage was set to 10 kV. Scans using SEM are usually acquired first at lower magnification (e.g., 100-microm scan) and the smaller areas are scanned within the larger area (30 micron, 10 micron).

The elemental composition was measured by energy-dispersive X-ray spectroscopy (EDS, analyzer X-ManN, a 20 mm^2^ SDD detector, Oxford Instruments, Buckinghamshire, United Kingdom), while the accelerating voltage for SEM-EDS analysis was set to 10 kV.

#### 3.3.2. Atomic Force Microscopy

Surface morphology and roughness of the pristine and laser-treated films were examined using the atomic force microscopy (AFM) technique using Dimension ICON (Bruker Corp., Billerica, MA, USA). The samples were analyzed in the Scan-Assyst^®^ mode using the nitride lever SCANASYST-AIR with a Si tip (a spring constant of 0.4 N·m^−1^). NanoScope Analysis software (version 1.80). was applied for data processing. Surface roughness (*R_a_*) represents the arithmetic mean of the absolute values of the height deviations measured from the central plane.

#### 3.3.3. X-Ray Photoelectron Spectrometry

The elemental composition on the material surface was analyzed by X-ray photoelectron spectroscopy (XPS) using a spectrometer, ESCAProbeP (Omicron Nanotechnology Ltd., Taunusstein, Germany). As a source, a monochromatic X-ray at an energy of 1486.7 eV was used. Atomic concentrations of elements were determined from the individual peak areas using CasaXPS software 2.3.17PR 1.1.

### 3.4. Evaluation of Antibacterial Activity

The antibacterial activity of pristine and laser-treated samples was measured using the drop plate method against two bacterial strains: the Gram-positive and Gram-negative bacteria *Staphylococcus epidermidis* (DBM 2124) and *Escherichia coli* (DBM 3138), respectively. Overnight cultures (16 h) of both strains were grown in Luria–Bertani (LB; Merck, USA) medium at 37 °C with shaking at 200 rpm. The optical density at 600 nm (OD_600_) of each culture was measured and adjusted to OD_600_ = 1 using prewarmed (37 °C) phosphate-buffered saline (PBS). The bacterial suspensions were then further diluted to 4 × 10^4^ and 2 × 10^4^ colony-forming units (CFU) per mL for *S. epidermidis* and *E. coli*, respectively. Aliquots of these suspensions were incubated with the tested samples at 37 °C for 3 and 6 h. Then, 25 μL drops were pipetted onto LB agar plates (5 drops per sample, three replicates) and incubated at 37 °C for 24 h. The resulting CFU numbers on plates from each sample were determined and averaged. Bacterial suspensions incubated in PBS without samples served as controls.

### 3.5. Cytocompatibility Evaluation

The cytocompatibility of the tested samples was evaluated using human osteosarcoma cells (U-2 OS; ATCC, Manassas, VA, USA). Cells were cultured at 37 °C, 5% CO_2_, and a humidified atmosphere in high-glucose Dulbecco’s modified Eagle’s medium (DMEM; Merck, Burlington, MA, USA) with 10% fetal bovine serum (FBS; Merck, USA). Cells were passaged three times per week using trypsin-EDTA and maintained in the exponential growth phase.

For cytocompatibility assessment, pristine and laser-treated samples were first placed into 24-well tissue culture plates (VWR/Avantor, Radnor, PA, USA), with each containing 1 mL of DMEM with 10% FBS, and incubated for 48 h under standard cultivation conditions to obtain material leachates. After incubation, the leachates were collected and used for subsequent cell viability assays.

U-2 OS cells were seeded at a density of 5000 cells per well into 96-well plates in 100 µL of DMEM with 10% FBS and enabled to grow for 24 h under standard cultivation conditions. Then, the cells were treated with 100 µL of the respective material leachates, and the cells were incubated for an additional 24, 48, or 72 h. After each incubation period, the medium was removed and replaced with 100 µL of phenol-red free DMEM (Merck, USA) containing the WST-1 reagent (4% *v*/*v*). Following a 1 h incubation, the absorbance of the resulting formazan product was measured at 450 nm with a background reference at 650 nm. Wells with cells cultured in standard medium without exposure to material leachates served as controls.

## 4. Conclusions

In this study, high-excimer laser processing was demonstrated as an effective strategy for tailoring the surface properties of TiGr5 ELI, TiNbZr, and TiNbSnTa alloys. The main aim was to assess the effect of these changes on antibacterial activity and cytocompatibility. Laser irradiation at a fluence of 1000 mJ·cm^−2^ with 200 pulses induced pronounced modifications in surface morphology and chemistry, as confirmed by AFM, SEM, and XPS analyses. The microdomain patterns on all three studied substrates was induced, with significant changes in both surface morphology and effective surface area (an increase in at least one of these values) compared to pristine couterparts. Beyond topographical restructuring, high-energy laser treatment caused marked changes in surface chemistry, most notably a significant enrichment of oxygen on the irradiated surfaces, which was confirmed by both EDS and XPS analyses. The combined effects of altered surface morphology and chemistry and the creation of a semi-periodic lamellar structure played a key role in modulating the biological responses. All laser-treated Ti-based substrates exhibited good cytocompatibility comparable to or slightly higher than that of the control samples, indicating no adverse effect on U-2 OS cell viability. Notably, after 72 h, U-2 OS cells incubated with laser-modified TiGr5 ELI exhibited the highest cell viability among all laser-treated samples. In parallel, the modified surfaces displayed enhanced antibacterial performance against both Gram-negative and Gram-positive bacteria, particularly against *S. epidermidis*. The programmable excimer exposure with steps in selected matrix also opens a possibility of large scale manufacturing of treated substrates. These results indicate that excimer laser-induced surface functionalization can simultaneously promote mammalian-cell viability while suppressing bacterial adhesion and growth, addressing a key challenge in implant material design.

## Data Availability

The data presented in this study are available at https://doi.org/10.5281/zenodo.21851371.

## Supplementary Materials

The following supporting information can be downloaded at https://www.mdpi.com/article/10.3390/ijms27167250/s1.
