# Supplementary material for: Effect of High-Energy Excimer Treatment of Ti-Based Alloys on Cytocompatibility and Antibacterial Properties
Source: Int J Mol Sci. 2026 Aug 14;27(16):7250. doi: 10.3390/ijms27167250 (PMC13513215; doi:10.3390/ijms27167250)
Supplement: Supplementary file 1 [file ijms-27-07250-s001.zip › ijms-4446907-supplementary.pdf]

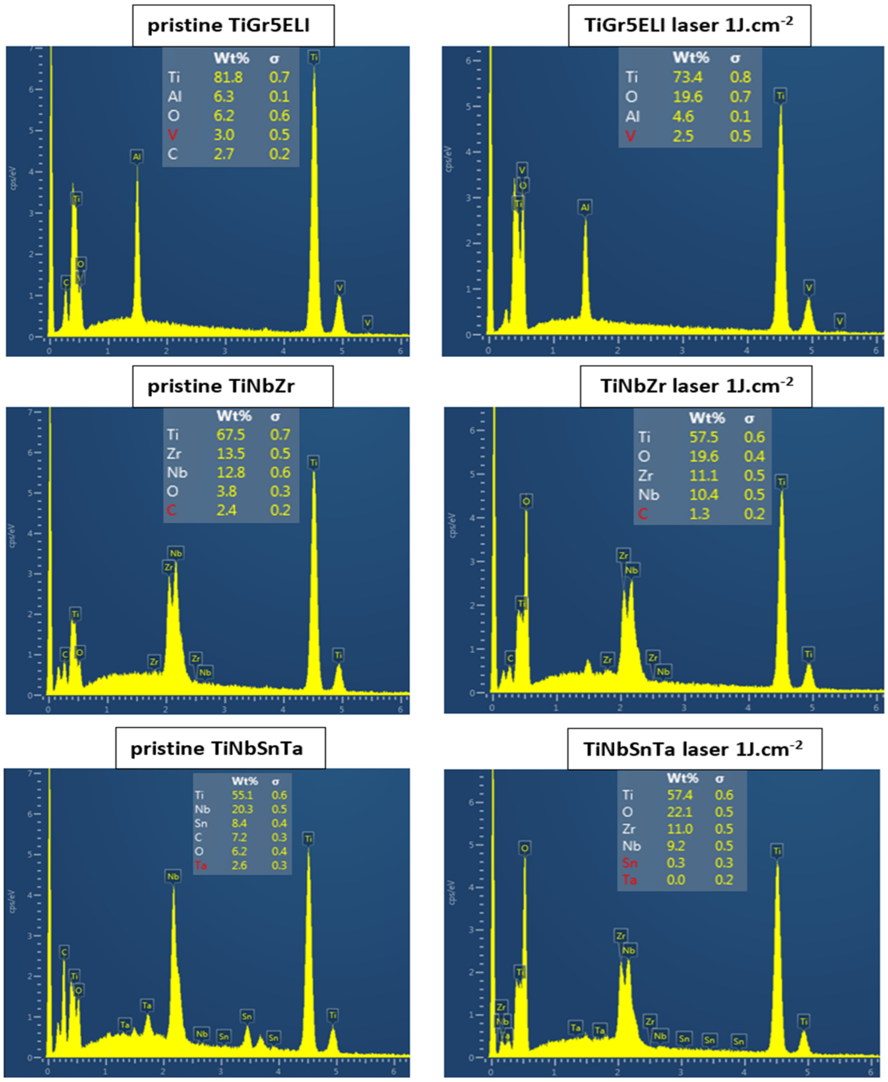

Figure S1. EDS spectra of pristine TiGr5 ELI, TiNbZr, and TiNbSnTa (left column) and these substrates after high-energy laser exposure with 1 J·cm^{-2} and 200 pulses (right column). At each spectrum, the weight concentration of particular elements is introduced.
